# Genome Sequence of Providencia rettgeri NVIT03, Isolated from Nasonia vitripennis

**DOI:** 10.1128/MRA.01157-18

**Published:** 2019-01-17

**Authors:** Guan-Hong Wang, Robert M. Brucker

**Affiliations:** a The Rowland Institute at Harvard University, Harvard University, Cambridge, Massachusetts, USA; University of Maryland School of Medicine

## Abstract

Providencia rettgeri is a common insect-associated Gram-negative bacterium. Here, we present the draft genome sequence of P. rettgeri NVIT03, the most common bacterial symbiont of the insect hymenopteran model Nasonia vitripennis. This symbiont is also part of the Sarcophaga bullata pupal microbiome that Nasonia spp. parasitize and that critically influences the development of the wasp.

## Abstract (precis)

Providencia rettgeri is a common insect-associated Gram-negative bacterium. Here, we present the draft genome sequence of P. rettgeri NVIT03, the most common bacterial symbiont of the insect hymenopteran model Nasonia vitripennis.

## ANNOUNCEMENT

The motile Gram-negative bacillus Providencia rettgeri was previously identified as a member of the gut microbiome of nematodes, insects, birds, and humans (1) while also being associated with opportunistic infections in humans and insects (2, 3). P. rettgeri is found throughout development in the dipteran genus Sarcophaga, a carrion-breeding necrophagous insect (4). These same insects serve as a host for the parasitic wasps of the genus Nasonia, which also maintain P. rettgeri in their gut microbial communities throughout development (5). P. rettgeri is the most dominant bacterium (67.00%) in the Nasonia vitripennis microbiome (5). This genome will help elucidate host-microbe interactions that are important to the affected insects’ physiology, development, behavior, reproduction, and evolution (6, 7).

We present here a draft genome sequence of P. rettgeri NVIT03, a gut bacterium isolated from the N. vitripennis strain AsymCx. After surface sterilizing whole *Nasonia* animals with 10% bleach for 2 min and then homogenizing them in sterile phosphate-buffered saline (PBS), we plated 1× aliquots of the homogenate onto brain heart infusion agar medium and confirmed the taxonomic identity by Sanger sequencing of the 16S rRNA before sending the isolate from the AsymCx strain for whole-genome sequencing.

Bacterial genomic DNA was isolated from P. rettgeri NVIT03 cells cultured in 100 ml of nutrient broth medium (catalog no. 1.05443.0500; Sigma-Aldrich) at 30°C and 250 rpm for 18 h in a flask. The culture was centrifuged at 6,000 rpm for 5 min, and the resulting cell pellet was used for genome extraction using the DNeasy blood and tissue kit (Qiagen, Hilden, Germany). DNA was quantified using the double-stranded DNA (dsDNA) high-sensitivity (HS) assay kit on the Qubit 2.0 fluorometer (Life Technologies, Waltham, MA, USA).

Library preparation and genome sequencing were performed at MicrobesNG (Birmingham, United Kingdom). The DNA library was prepared using the Nextera XT library prep kit (Illumina) and sequenced on the Illumina HiSeq platform using a 250-bp paired-end protocol. Sequencing resulted in 191,241 reads which were trimmed using Trimmomatic 0.36 (8) and quality filtered using SAMtools (9), BEDTools (10), and bwa-mem (11). Seventy-three contigs were assembled with SPAdes (version 3.8) (12), with an *N*_50_ value of 787,417 bp, 4,400,855 nucleotides in total, and about 164-fold coverage. This strain is most closely related to the previously sequenced P. rettgeri strain AR_0082 (GenBank accession no. CP029736). The genome of P. rettgeri NVIT03 consists presumably of a single chromosome (4,380,957 bp) and exhibits a G+C content of 40.30%. No evidence of plasmids was found based on analysis with PlasmidFinder version 1.3 (13). Annotation was performed using Prokka 1.12 (14). The genome contains 4,097 predicted protein-encoding genes with deduced functions and 1,242 genes (30.31%) coding for hypothetical proteins. Coding genes were identified using RNAmmer (15) and tRNAscan (16). The draft genome encodes 8 rRNAs and 72 tRNAs.

Using the CosmosID package (CosmosID, Inc., Rockville, MD, USA), no putative virulence factors were identified from P. rettgeri NVIT03. As the dominant bacterium of the microbiome of *Nasonia* spp., annotation of the P. rettgeri genome increases our insight into the mechanisms of this host-bacterium association.

### Data availability.

This whole-genome shotgun project has been deposited at DDBJ/ENA/GenBank under the accession no. QUAF00000000. The version described in this paper is version QUAF01000000. The BioProject number is PRJNA484797.
